# The cooking task: making a meal of executive functions

**DOI:** 10.3389/fnbeh.2015.00022

**Published:** 2015-02-11

**Authors:** T. A. Doherty, L. A. Barker, R. Denniss, A. Jalil, M. D. Beer

**Affiliations:** ^1^Brain Behaviour and Cognition Group, Department of Psychology, Sociology and Politics, Sheffield Hallam UniversitySheffield, UK; ^2^Communication and Computing Research Centre (CCRC), Sheffield Hallam UniversitySheffield, UK

**Keywords:** executive function, head injury, ecological validity, cooking task, neuropsychological assessment

## Abstract

Current standardized neuropsychological tests may fail to accurately capture real-world executive deficits. We developed a computer-based Cooking Task (CT) assessment of executive functions and trialed the measure with a normative group before use with a head-injured population. Forty-six participants completed the computerized CT and subtests from standardized neuropsychological tasks, including the Tower and Sorting Tests of executive function from the Delis-Kaplan Executive Function System (D-KEFS) and the Cambridge prospective memory test (CAMPROMPT), in order to examine whether standardized executive function tasks, predicted performance on measurement indices from the CT. Findings showed that verbal comprehension, rule detection and prospective memory contributed to measures of prospective planning accuracy and strategy implementation of the CT. Results also showed that functions necessary for cooking efficacy differ as an effect of task demands (difficulty levels). Performance on rule detection, strategy implementation and flexible thinking executive function measures contributed to accuracy on the CT. These findings raise questions about the functions captured by present standardized tasks particularly at varying levels of difficulty and during dual-task performance. Our preliminary findings also indicate that CT measures can effectively distinguish between executive function and Full Scale IQ abilities. Results of the present study indicate that the CT shows promise as an ecologically valid measure of executive function for future use with a head-injured population and indexes selective executive function’s captured by standardized tests.

## Introduction

Executive functions are higher-order cognitive processes associated with frontal brain networks essential for goal-directed behavior and include planning, temporal sequencing, and goal-attainment functions (Shallice and Burgess, 1991; Miyake et al., 2000; Royall et al., 2002; Barker et al., 2010; Morton and Barker, 2010). Individuals with frontal pathology often show diminished planning, self-correction, goal attainment and decision making abilities thought to be important for “real world” activities of daily living (ADL’s—Grafman et al., 1993; Godbout and Doyon, 1995; Godbout et al., 2005; Burgess et al., 2006). Consequently, executive function deficits may result in difficulty performing everyday tasks including shopping (Shallice et al., 1989; Shallice and Burgess, 1991), cooking a meal (Godbout et al., 2005), and simple tasks such as teeth brushing (Schwartz et al., 1998). However, research suggests that current executive function tasks have limited ability to predict ADL’s (Eslinger and Damasio, 1985; Burgess et al., 2006; Chan et al., 2008). Similarly, there are several reported cases with frontal pathology and normal scores on executive function tests, but diminished capacity to engage in ADL’s, suggesting that standard tests do not reliably capture “real world” problems (Shallice and Burgess, 1991; Chevignard et al., 2000; Andrés and Van der Linden, 2002; Barker et al., 2004).

The act of cooking a meal requires several executive functions, including capacity to multitask, plan, use prospective memory and maintain and complete, both sub and overall goals within a strict timeframe (Craik and Bialystok, 2006). Although there is limited research, previous findings suggest that cooking tasks (CT) may be more sensitive to patient deficits than traditional neuropsychological measures (Chevignard et al., 2000, 2008; Fortin et al., 2003; Craik and Bialystok, 2006; Tanguay et al., 2014). Fortin et al. (2003) found no difference between a head-injured group and controls on standardized assessment, although the patient group showed diminished ability to cook a meal. The authors concluded that impaired planning and prospective memory functions contributed to diminished ability to cook a meal in the patient group and these deficits were not captured by standardized tests. Chevignard et al. (2008) compared performance of brain injured participants and controls on a semi-structured CT conducted in the occupational therapy kitchen and standardized measures of executive function. Patients made numerous errors, including context neglect, purposeless action and environmental adherence indicating abnormal responses to contextual and environmental cues. Cooking performance variables, including number of errors, cooking duration, goal achievement, and dangerous behaviors were all predicted by the Six Elements Task, a standardized version of the Multiple Errands Test (Shallice and Burgess, 1991; Wilson et al., 1997), indicating that their CT and an ecologically derived executive function measure, indexed similar functions in contrast to findings of Fortin et al. (2003). Kerr (unpublished) and Craik and Bialystok (2006) developed a CT to investigate planning ability in an elderly population and found that the task was sensitive to aging effects. However, task indices only weakly correlated with scores on standardized measures. They concluded that CT are potentially useful laboratory based methods of planning corresponding well to real world ADL’s.

Previous findings indicate that cooking can provide a sensitive and reliable measure of executive function ability in a “real-world” context. However, “real” CT require elaborate setup, are time consuming and require ongoing monitoring of the individual’s progress that is not easily standardized, for later follow-up or across group comparisons. Hence, a compromise must be made between unrealistic conditions of lab-based assessment and “real-world, real-time” tasks that are time costly and difficult to replicate when developing an ecologically valid task for clinical assessment and guiding rehabilitation programs. The core components of the real-life task should be captured by the ecologically valid version and be sufficiently standardized that performance can be compared across time points at follow up and across neuropathological groups. Additionally, mixed findings of previous research renders it difficult to establish whether “real-world” cooking ability corresponds to executive functions indexed by standardized clinical measures.

With this aim in mind the current study employed a computer-based simulation of cooking a meal based on the CT developed by Kerr (unpublished) and Craik and Bialystok (2006). The present CT shares some similarities with the original including a comparative user interface and secondary distracter task of table setting as well as copious modifications. In the current task, ability to pause an item whilst it was cooking was seen as a necessity; in real-world settings individuals can stop items cooking if they believe they have initiated cooking at the wrong time. This mid-plan adjustment seemed necessary to document as it increased the sensitivity of the measure beyond whether the end goal was completed or not. Additionally, the original task by Craik and Bialystok (2006) had no variety in the number of items to cook, simply the number of screens on which these items were presented. In the interests of maintaining ecological validity this screen-switching was dropped in favor of different difficulty levels pertaining to the number of items that required cooking within a set time frame, and whether or not setting the table was necessary. The current task also provided more detailed measures, which were calculated by the program itself. The present study compared indices of our newly developed computerized CT with standardized neuropsychological tasks thought to relate to cooking a meal, including measures of planning, prospective memory, and temporal sequencing in a normal population.

## Method

### Participants

Table 1 shows the broad age range (early adulthood through to older adults) and the variability in Full Scale IQ, ranging from Low Average to Superior, of the forty-six participants, which is indicative of the diverse nature of the normative sample in the present study.

**Table 1:** **Demographic data of participants (*n* = 46)**.

Demographic	Mean (SD) [Range]
Age	29.50 (9.55) [18–59]
Full scale IQ (FSIQ)	111.46 (12.20) [83–130]
Years of education	14 (1.90) [11–17]
Gender split (M/F)	21/26

All participants gave their informed consent and the faculty research ethics board approved the research. We sampled participants from a broad demography to test the sensitivity of the task within a diverse sample. Participants completed three standardized executive function tests, a measure of IQ and the computerized CT in a laboratory setting. One of the main reasons for the present study, was to test the computational viability of our new CT, whether a new shortened version accurately indexed executive functions measured by selected tasks from our battery of tests and whether these functions were sensitively captured by the task with a small group of non-neuropathological controls, before trialing the task with a brain-injured cohort. Tests were administered in one session in counterbalanced order with participant-determined rest breaks. We selected executive function tests thought to contribute to real world cooking ability (Chevignard et al., 2008), or previously shown to be associated with ability on our earlier version of the CT (McFarquhar and Barker, 2012).

### The cooking task

The present task shares some similarities with an earlier task developed by Craik and Lockheart (2006) including a comparative user interface and secondary distracter task of table setting. In real-world settings individuals can stop items cooking if they believe they have initiated cooking at the wrong time. This mid-plan adjustment was important to measure because it increased task sensitivity and provided an index of prospective plan accuracy. Various adaptations were made to the original version of the CT in the present study to account for data collection with a non-neuropathological group and to shorten testing time, which was originally 3 h duration (McFarquhar and Barker, 2012). The computational design of the CT was a lengthy process because we wanted to generate a measure that was as similar as possible to “real-world” behavior whilst maintaining clear measurement indices and a relatively interactive user-interface. The development of the CT program will not be discussed further here except in relation to the measurement variables generated by the task, participant instructions and the appearance of the task.

The CT was programed using MIT App Inventor version 1.34 (M.I.T., 2014) and built for devices running the android operating system. The task was administered on a 16 Gb, 1.5 Ghz Quad Core Asus Google Nexus 7’ tablet computer running Android OS 4.4 (KitKat). At start-up the task displayed a welcome screen and a keypress button for the “Instructions” screen. Participants were required to read the instructions carefully before returning to the welcome screen and proceeding to the first level of the task. The task comprised four levels (two tasks per level) and participants had to successfully complete each level before moving onto the next level. A task fail occurred if some items were not cooked within the given time frame, or if any of the items were left to “go cold” (are left cooked for 30 sec), whilst other items were cooking. Participants were permitted a second attempt if they failed the first task on a level; only the food items changed with no change in the task parameters from the first task to the second task at the same level. The first screen of each level of the CT informed participants of time available for task completion. The next screen was the planning screen loaded by pressing the “start planning” button and presented information on how many items to cook at that level and a keypress button to re-load the Instruction screen if needed. The planning screen presented the relevant cooking times for each item as well as a brief reminder of the relevant rules for successfully completing the task.

**Figure 1:**
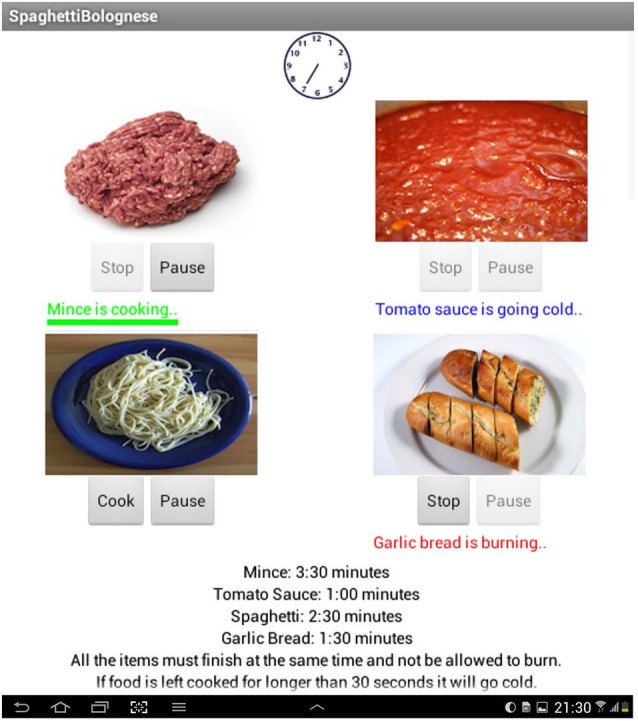
**A screenshot from the main screen of the Bolognese task at the “difficult” level with secondary laying the table distracter: (from left to right) a paused item, a finished item, a burning item, a cooking item and an item that has not been started**.

Food items were represented by an image with *“cook”* (which changed to *“stop”* once pressed however, the button remains inactive as an item can only be stopped once it has been fully cooked) and *“pause”* (which changed to *“resume”* once pressed) buttons below the image. Whilst the item cooked a timer bar (which reduced at a proportional rate to the length of time the item cooked for) was green, with the text stating “^*^item^*^ is cooking”, when the item cooked over the allotted time the timer bar disappeared and red text stated “^*^item^*^ is burning”. Finally when the item cooking was stopped a blue text message stated “^*^item^*^ is going cold”. Sound files for each item loaded during cooking time (recorded from real cooking of these items) in order to simulate a real world analog auditory prompt for the participant and improve the ecological validity of the task (McGuire, 2014). The cooking time for each item was presented at the bottom of the screen along with information outlining the basic parameters of the task and a “real time” clock present on each task screen (see Figure 1).

The data recorded during the task was cooking time for each item, burning time for each item (time left cooking over the suggested time), pause time for each item, time each item is left cold for and the remaining amount of allotted time for each task.

**Figure 2:**
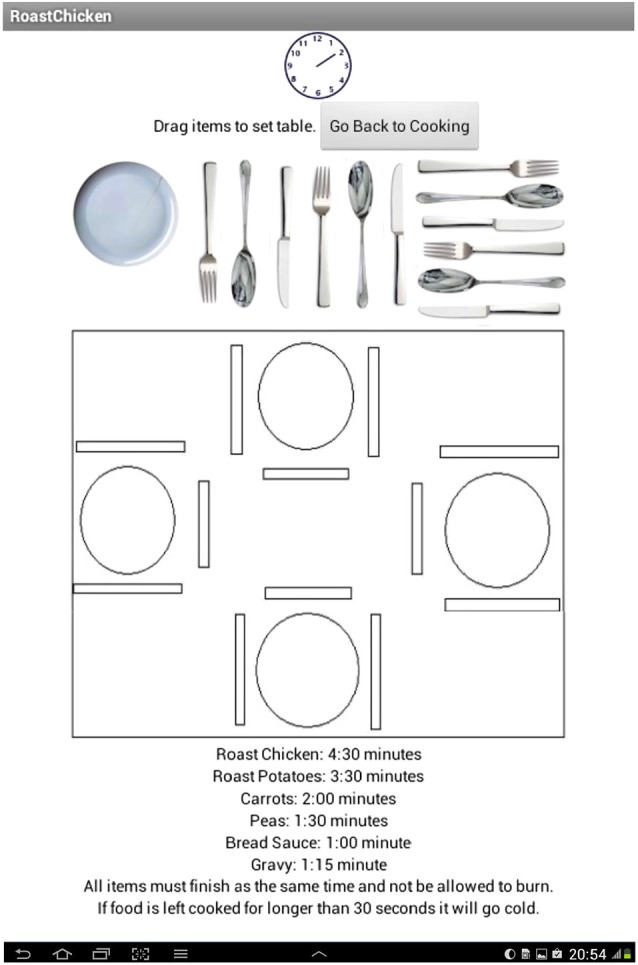
**Table setting with the bank of items (plates; knives; forks and spoons)**.

Level one of the CT required participants to cook two items within 2 min (Easy level), level two consisted of four items to cook within 4 min of cooking time (Moderate level), level three consisted of six items to be cooked within 5 min (Difficult level) and level four required participants to cook six items within 5 min and included a separate distracter task where the participant must lay a virtual table concurrent with the CT (Dual-task level). During the Dual-task level of the task, the screen additionally included an image of a dining table and crockery items. To lay the table participants were required to drag and drop items (a fork, knife, spoon and plate) to each of four empty table settings (participants could switch between this task and the primary CT for the task duration they had to complete the task however within the overall 5 min duration—see Figure 2). Performance on the secondary task was scored on a pass-fail basis. The time to complete the CT, ranged between 17–35 min, but mostly took under 20 min to complete in this cohort of healthy controls.

Upon successful completion of each task a “congratulations” screen appeared with a keypress button that loaded the next level of the task. However, if the task was failed the program loaded a screen detailing why the task was failed and a reminder of rules transgressed during task completion. This screen also displayed a keypress button that loaded the second task of that level, or if a task on that level was failed twice a goodbye screen was loaded and the CT was completed.

### Outcome measures generated by the cooking task

During performance on the task the computer program recorded times for each level and burn time, pause time, cold time and remaining time for all items as well as an overall accuracy ratio. These raw data were then transformed into three specific variables based on scores originally used by Craik and Bialystok (2006) and used in our earlier computerized CT: Range, Discrepancy and Adjustment scores (McFarquhar and Barker, 2012).

### Range score: measure of time-based strategy use

The Range score calculated the difference between the time the first item was stopped and the last item was stopped. This provided a measure of prospective time-based strategy implementation and the value should therefore be close to zero. Much like a real world task it is impossible to stop all items at the same time however, this score accurately measures if a participant has forgotten a specific item, it is therefore calculated on an item-by-item basis and the highest scoring item is taken as the range score for that level.

### Discrepancy score: measure of prospective plan implementation

The Discrepancy score calculated the difference between the actual amount of time an item was cooked for, including the burn time, and the prescribed cooking time. An average of all item scores was calculated to give a score for each level. This value provided a measure of prospective plan accuracy: the ability to plan, implement and remember to start and stop all items at the correct time. This value should therefore be close to zero. Previous studies indicated this provides a measure of prospective memory, as participants need to remember to start and stop the items at the correct time.

### The adjustment score: measure of plan accuracy

The Adjustment score calculated the amount of time all the items were paused for. This provided a measure of plan accuracy as an accurate and effective plan should require no mid-task adjustments. This value should also therefore approach zero. Again an average of all item scores was calculated to provide a score for each level.

The CT program also generated two further variables in order to measure a participant’s comprehensive performance, both per level and as a task overall, on completed levels of the CT.

### The achievement score

A fourth measure, which was designed to be an achievement measure per level, was taken from the remaining time left from each level. This was calculated by dividing the number of items on the level by the time remaining for that level.

### The accuracy ratio

The final measure was an overall measure of accuracy, termed the accuracy ratio and was calculated by measuring the number of tasks attempted by a participant. We computed this measure in order to give an accuracy measurement of the number of levels/failed trials undertaken by a participant over the course of the entire task.

### Standardized IQ and executive function measures

#### WASI (Wechsler abbreviated scale of intelligence—Wechsler, 1999)

The WASI (Wechsler, 1999) was used to provide a measure of overall Full Scale and Verbal and Performance IQ scores and account for any potential individual differences that might affect scores on executive function and CT tests. We hypothesized that IQ subtests would predict strategy implementation (Range score) and prospective plan implementation (Discrepancy scores) of the CT, due to our previous findings from work with a lengthier and more time consuming version of the CT (McFarquhar and Barker, 2012). We also anticipated that Full Scale IQ would predict overall performance across all levels of the CT. Scores from the WASI have been found to produce reliability coefficients between *r* = 0.97 and *r* = 0.98.

#### D-KEFS—tower test (Delis-Kaplan Executive Function System—Delis et al., 2001)

We selected measures from the Delis-Kaplan Executive Function System (D-KEFS) executive function battery for the present study as these measures are widely used in clinical and academic work with neuropathological groups (Baldo et al., 2003; Martin et al., 2003) and have good levels of reliability and sensitivity. The Tower Test indexes planning accuracy and rule detection ability (Crawford et al., 2011) and also generates several composite scores that we expected to contribute to performance on the CT. Tower Task time per move ratio provides a measure of the average time an examinee takes to make each move throughout the task. According to the manual normative samples show consistency in time spent “pausing and studying moves”. We expected this variable to predict the CT measure of planning accuracy (Adjustment score). Tower Task rule violation per item ratio represents the number of rule breaks made over the course of all items. Thus this score provides a measure of rule detection ability; again we expected scores on this measure to predict CT planning accuracy scores (Adjustment scores), strategy implementation scores (Range scores) and prospective plan implementation score (Discrepancy scores) on the CT. The reliability co-efficient for total achievement score on the Tower Test is *r* = 0.44.

#### D-KEFS—sorting test (Delis et al., 2001)

The Sorting Test measures flexible thinking ability, concept formation (verbal and non-verbal) and strategy initiation. These functions are thought to play a role in the capacity to cook a meal (Chevignard et al., 2008). This test has been shown to require strategy initiation and capacity to inhibit pre-potent responses and is sensitive to performance differences between neuropathological groups and controls (Parmenter et al., 2007; Heled et al., 2012). The Sorting Test also generates several composite scores that we expected to contribute to performance on the CT; composite scaled score provides a measure of accuracy in sorting rules, or concepts across free sort and sort recognition conditions, thus it combines performance scores across both conditions of the Sorting Test. According to the manual high scores represent effective use of high-level executive function concept-formation/strategy generation rules. We anticipated that the Sorting Test composite score would predict strategy implementation (Range score) of the CT. Sorting Test contrast scaled score provides a calculation of the difference between an individual’s abilities to develop a sorting concept and describe that sorting concept providing an index of concept formation flexibility. We hypothesized that ability on these measures would predict planning accuracy (Adjustment scores) on the CT. Scores from the D-KEFS Sorting Test have been found to produce a reliability coefficient of *r* = 0.46 depending upon subtests used.

#### CAMPROMPT (Cambridge prospective memory test—Wilson et al., 2005)

We selected a standardized prospective memory task because previous research found a relationship between performance on an earlier version of the present CT and prospective memory scores on a nonstandardized task (McFarquhar and Barker, 2012). In the present study we wanted to investigate whether a relationship between CT measures and prospective memory scores remained when the standardized Cambridge prospective memory test (CAMPROMPT) task used in clinical settings was used. Scores from the CAMPROMPT have been found to produce a reliability coefficient of *r* = 0.64. We expected CAMPROMPT subtests to predict prospective strategy implementation (Range scores) on the CT.

## Results

All raw data were standardized using Z transformation to control for outliers and compare scores across neuropsychological and CT variables. Any outliers that exceeded 3.29 after transformation were excluded in line with recommendations for treatment of outliers in transformed datasets (Ratcliffe, 1993; Field, 2009). This included one case across each level of the CT mid-plan Adjustment variable. We also computed Pearson’s correlation analyses for our selected variables for each regression analyses to thoroughly explore data and check for multi-colinearity.

Table 2 presents descriptive data for the neuropsychological tests used in the present study.

**Table 2:** **Mean (SD) and [Range] values for standardized neuropsychological test variables (*N* = 46)**.

Neuropsychological measure	Mean (SD) [Range]
*WASI IQ measure*
Full scale IQ (FSIQ)	111.46 (12.20) [83–130]
Perceptual reasoning index (PRI)	113.41 (11.78) [87–138]
Verbal comprehension index (VCI)	106.91 (13.83) [71–132]
*CAMPROMPT measure*
Overall score	34.00 (1.74) [30–36]
Time based score	17.57 (0.84) [16–18]
Event based score	16.43 (1.52) [14–18]
*D-KEFS tower test*
Total accuracy score	11.85 (2.31) [8–17]
Mean first move time	10.96 (1.70) [7–14]
Time per move ratio	10.91 (1.28) [8–14]
Move accuracy ratio	9.78 (2.24) [5–13]
Rule violation per item ratio	10.20 (1.20) [3–11]
*D-KEFS sorting test*
Confirmed correct sorts	12.54 (2.43) [8–17]
Free sorting description score	11.72 (2.51) [7–16]
Sort recognition description score	13.04 (3.61) [3–18]
Composite scaled score	12.93 (3.19) [6–18]
Contrast scaled score	11.26 (2.33) [2–16]

Table 3 shows the CT variables.

**Table 3:** **Standardized (Z) scores: Mean (SD) and [Range] values, for easy, moderate, difficult and dual-task levels of the cooking task indices with outliers removed (*n* = 45 for adjustment variable across all levels, *n* = 46 for all other variables)**.

Cooking task variable	Level 1	Level 2	Level 3	Level 4
Range variable	0.00 (1.00) [−1.34–2.19]	0.08 (0.87) [−1.50–2.64]	0.00 (1.00) [−1.27–3.11]	0.21 (1.00) [−1.38–3.11]
Discrepancy score	0.00 (1.00) [−1.13–2.56]	0.00 (1.00) [−1.78–2.89]	0.00 (1.00) [−1.79–2.74]	0.00 (1.00) [−1.61–2.72]
Adjustment score	−0.07 (0.85) [−0.45–3.10]	−0.14 (0.23) [−0.22–0.84]	−0.10 (0.74) [−0.44–2.30]	−0.07 (0.87) [−0.64–2.8]
Residual Time	0.00 (1.00) [−1.13–2.56]	0.00 (1.00) [−1.78–2.89]	0.00 (1.00) [−1.79–2.74]	0.00 (1.00) [−1.61–2.72]
Table items set (n/16)		n/a		15.89 (0.74) [11–16]
Level accuracy ratio (%)	90.09 (12.02) [66–100]			

Results of One-Way ANOVA for Range scores across different levels of the CT showed that performance was significantly different for this measure of time-based strategy implementation *F*_(3,183)_ = 21.9, *p* = 0.00. Similarly, performance was significantly different for Discrepancy scores (measure of prospective plan implementation) across levels of task difficulty *F*_(3,183)_ = 15.2, *p* = 0.00. Scores were also significantly different for the Adjustment variable (measure of plan accuracy) across different task levels *F*_(3,178)_ = 4.74, *p* = 0.00. Table 4 shows results of Tukey HSD *post hoc* analyses for comparison between each difficulty level for each CT measure. For the Range variable (measure of time-based strategy implementation) performance was different across all levels except for 3 and 4, for Discrepancy score (measure of prospective plan implementation) levels 1 and 3 and 1 and 4 were different, 2 and 3 and 2 and 4 were different and 1 and 2 and 3 and 4 were not different. For Adjustment score (measure of plan accuracy) levels 1 and 4, and 3 and 4 were different (see Table 4).

**Table 4:** **Results of Tukey HSD *post hoc* analyses across all difficulty levels (1–4), for range, discrepancy and adjustment measures of the cooking task**.

Cooking task difficulty levels		Range score: mean dif. (sig.)	Discrepancy score: mean dif. (sig.)	Adjustment score: mean dif. (sig.)
1	2	−2122.6 (*p* = 0.01)	−131.0 (*p* = 0.92)	−1528.6 (*p* = 0.80)
	3	−4100.2 (*p* = 0.00)	−1044.3 (*p* = 0.00)	−308.4 (*p* = 0.99)
	4	−5007.9 (*p* = 0.00)	−1004.0 (*p* = 0.00)	−5680.3 (*p* = 0.00)
2	1	2122.6 (*p* = 0.01)	131.0 (*p* = 0.92)	1528.6 (*p* = 0.80)
	3	−1977.5 (*p* = 0.01)	−913.3 (*p* = 0.00)	1220.2 (*p* = 0.89)
	4	−2885.3 (*p* = 0.00)	−873.0 (*p* = 0.00)	−4151.7 (*p* = 0.07)
3	1	4100.2 (*p* = 0.00)	1044.3 (*p* = 0.00)	308.4 (*p* = 0.99)
	2	1977.5 (*p* = 0.01)	913.3 (*p* = 0.00)	−1220.2 (*p* = 0.89)
	4	−907.7 (*p* = 0.53)	40.3 (*p* = 0.99)	−5371.9 (*p* = 0.01)
4	1	5007.9 (*p* = 0.00)	1004.0 (*p* = 0.00)	5680.3 (*p* = 0.00)
	2	2885.3 (*p* = 0.00)	873.0 (*p* = 0.00)	4151.7 (*p* = 0.07)
	3	907.7 (*p* = 0.53)	−40.3 (*p* = 0.99)	5371.9 (*p* = 0.01)

We developed predictor models on the basis of functions purportedly tapped by neuropsychological and corresponding CT variables as outlined previously. All reported significance levels are one-tailed due to our *apriori* hypotheses. We analyzed each CT level (levels 1–4) separately to establish whether the pattern of relationships between variables differed as an effect of level difficulty.

### Range score: a measure of time-based strategy implementation

We entered Event Based Scores from the CAMPROMPT (episodic prospective memory; *r* = 0.14, *p* = 0.35), Perceptual Reasoning Index of the WASI (performance IQ; *r* = −0.28, *p* = 0.053), Tower Test Rule Violation Per Item Ratio (rule detection; *r* = −0.05, *p* = 0.69) and Sorting Test Confirmed Correct Sorts (concept formation; *r* = −0.25, *p* = 0.08). We expected performance on these measures to contribute to effective time-based strategy implementation. Results of Pearson’s correlation showed a weak negative relationship between performance IQ, concept formation and Range 1 scores. For the easy level (Range 1), the model was not significant *F*_(5,45)_ = 1.13, *p* > 0.05, and the only marginally significant predictor was performance IQ of the WASI (*β* = −0.25, *p* = 0.06). Results of Pearson’s correlations for the moderate difficulty level (Range 2) showed only a moderate relationship between episodic prospective memory *r* = −0.30, *p* = 0.40 and Range 2 scores (performance IQ; *r* = 0.03, *p* = 0.80, rule detection; *r* = 0.04, *p* = 0.80 and concept formation; *r* = 0.02, *p* = 0.90). The model was not significant *F*_(5,45)_ = 0.90, *p* > 0.1. However, episodic prospective memory was a significant predictor of Range 2 scores (*β* = −0.31, *p* = 0.02). For the difficult level results of Pearson’s correlations showed a weak negative relationship between rule detection *r* = −0.28, *p* = 0.052 and Range 3 scores (episodic prospective memory; *r* = 0.05, *p* = 0.72, performance IQ; *r* = −0.02, *p* = 0.91, rule detection; *r* = −0.28, *p* = 0.052 and concept formation; *r* = 0.06, *p* = 0.68). Again the model was not significant *F*_(5,45)_ = 1.31, *p* > 0.05, and rule detection was the only significant predictor of Range 3 scores, (*β* = −2.31, *p* = 0.01). Finally, at the dual-task level results of Pearson’s correlations showed only a weak negative correlation (*r* = −0.26, *p* = 0.07) between concept formation and Range 4 scores (rule detection; *r* = −0.05, *p* = 0.73, episodic prospective memory; *r* = −0.11, *p* = 0.44, and performance IQ; *r* = −0.05, *p* = 0.73). The model was not significant *F*_(5,45)_ = 1.20, *p* > 0.05, although concept formation (*β* = −2.13, *p* = 0.01), and episodic prospective memory (*β* = −1.43, *p* = 0.05) predicted Range scores at the dual-task level.

### Discrepancy scores: prospective plan implementation

Discrepancy scores on the CT represent ability to implement and follow a plan for accurate start and stop times for all CT stimulus items for each difficulty level. We anticipated that verbal working memory might contribute to plan generation and implementation and prospective memory indexed by the CAMPROMPT. So we entered the Vocabulary Comprehension Index (VCI—Verbal IQ) of the WASI and Event Based scores of the CAMPROMPT as predictors in the model with discrepancy scores as the criterion variable for each difficulty level. Results of Pearson’s correlations were not significant for Verbal IQ (*r* = −0.13, *p* = 0.40) and prospective memory (*r* = −0.17, *p* = 0.26) and Discrepancy 1 scores. The model was not significant at the easy level (Discrepancy 1) *F*_(2,45)_ = 1.01, *p* > 0.05 and prospective memory scores marginally predicted discrepancy scores (*β* = −1.23, *p* = 0.07). At the medium difficulty level results of Pearson’s correlations showed a weak positive relationship (*r* = 0.33, *p* = 0.02) between prospective memory and Discrepancy 2 scores, but not for Verbal IQ and Discrepancy 2 scores (*r* = 0.02, *p* = 0.91). The model was significant at this level *F*_(2,45)_ = 4.10, *p* < 0.01 and prospective memory (*β* = 0.34, *p* < 0.01) and Verbal IQ scores (*β* = 0.22, *p* = 0.05) predicted Discrepancy 2 scores. Results of Pearson’s correlations at the difficult level showed a weak negative moderate correlation between Verbal IQ and Discrepancy 3 scores (*r* = −0.29, *p* = 0.054) and a weak relationship between prospective memory and Discrepancy 3 scores (*r* = 0.21, *p* = 0.16). At this level the model was not significant *F*_(2,45)_ = 1.96, *p* = 0.06, and prospective memory (*β* = 0.21, *p* = 0.06) and Verbal IQ (*β* = −0.19, *p* > 0.05) scores only marginally predicted the criterion variable. At the dual-task level results of Pearson’s correlations showed a weak negative relationship between Verbal IQ and Discrepancy 4 scores (*r* = −0.22, *p* = 0.14), not shown for prospective memory and Discrepancy 4 scores (*r* = 0.04, *p* = 0.78). The regression model was significant at the dual-task level *F*_(2,45)_ = 2.63, *p* < 0.05, and Verbal IQ score was the unique predictor of Discrepancy score (*β* = −0.32, *p* < 0.05) at this level.

### Adjustment scores: a measure of plan accuracy

Adjustment scores of the CT arguably measure the ability to generate an accurate plan. We hypothesized that performance on the Tower and Sorting tests would predict Adjustment scores because these indices capture components of planning likely to contribute to prospective plan generation for synchronous cooking of CT stimulus (food) items. We entered Tower Test Time Per Move Ratio (time-based plan accuracy and implementation), Tower Test Rule Violations (rule detection) and Sorting Test Contrast Score (flexible thinking). Results of Pearson’s correlations for time based plan accuracy (*r* = 0.15, *p* = 0.92), rule detection (*r* = −0.30, *p* = 0.05) and flexible thinking (*r* = 0.16, *p* = 0.28) showed only a weak relationship between rule detection and adjustment score at the easy level. The regression model was not significant for the easy level *F*_(3,44)_ = 1.87, *p* > 0.05 and rule detection score was the only significant predictor of Adjustment 1 scores (*β* = 0.30, *p* < 0.05). At the moderate level of the task, results of Pearson’s correlation showed a very weak relationship between plan accuracy (*r* = 0.04, *p* = 0.78), rule detection (*r* = −0.01, *p* = 0.99) and flexible thinking (*r* = 0.08, *p* = 0.61) and Adjustment 2 scores. The regression model was not significant for the moderate level of the task *F*_(3,44)_ = 0.15, *p* > 0.05 and none of the variables predicted performance on Adjustment 2 scores. At the difficult level, results of Pearson’s correlation showed a weak relationship between flexible thinking (*r* = 0.32, *p* = 0.03) and Adjustment 3 scores, but plan accuracy (*r* = 0.21, *p* = 0.17) and rule detection scores did not significantly correlate with Adjustment 3 scores. The regression model was not significant *F*_(3,44)_ = 1.59, *p* > 0.05 although flexible thinking was a significant predictor (*β* = −0.29, *p* < 0.05) of Adjustment 3 scores.

Finally, at the dual-task level there was a significant negative moderate relationship between rule detection (*r* = −0.40, *p* = 0.01) and Adjustment 4 scores, not present for plan accuracy (*r* = −0.16, *p* = 0.29) or flexible thinking (*r* = 0.17, *p* = 0.25) and Adjustment scores. The regression model was significant at this level *F*_(4,45)_ = 2.90, *p* > 0.01, and rule detection score was the only significant predictor of plan accuracy (Adjustment 4) at this level.

### Residual time: a measure of task accuracy

We entered Sorting Test Recognition Description Score (verbal concept formation), Sorting Test Composite Score (strategy initiation) and Tower Test Rule Violation Per Item (rule detection) as predictors.

Results of Pearson’s correlations for verbal concept formation (*r* = 0.04, *p* = 0.79), strategy initiation (*r* = 0.20, *p* = 0.17) and rule detection (*r* = 0.15, *p* = 0.30) showed a moderate relationship between rule detection and residual time at the easy level. The regression model was significant at this level *F*_(3,45)_ = 3.10, *p* < 0.05, and rule detection was the only significant predictor (*β* = 0.38, *p* < 0.01) of the criterion variable. At the moderate task difficulty level, results of Pearson’s correlations for verbal concept formation (*r* = −0.07, *p* = 0.64), strategy initiation (*r* = 0.05, *p* = 0.74) and rule detection (*r* = 0.46, *p* = 0.00) again showed a moderate relationship between rule detection and residual time. The model was also significant at the moderate level *F*_(3,45)_ = 6.10, *p* < 0.01, and verbal concept formation (*β* = −0.80, *p* < 0.001), strategy initiation (*β* = 0.47, *p* < 0.001) and rule detection (*β* = 0.64, *p* < 0.05) were significant predictors of residual time at this level. Results of Pearson’s correlations for verbal concept formation (*r* = 0.18, *p* = 0.22), strategy initiation (*r* = 0.23, *p* = 0.12) and rule detection (*r* = 0.22, *p* = 0.14) showed only a weak relationship between EF variables and residual time left at the difficult level. The model was not significant at the difficult level of the CT *F*_(3,45)_ = 1.35, *p* > 0.05 and strategy initiation was the only significant predictor (*β* = 0.40, *p* = 0.05) of the criterion at this level. At the dual-task level, results of Pearson’s correlations for verbal concept formation (*r* = 0.04, *p* = 0.79), strategy initiation (*r* = −0.05, *p* = 0.75) and rule detection (*r* = 0.13, *p* = 0.39) showed only a very weak relationship between EF variables and residual time left. Similarly, at the dual-task level the model was not significant *F*_(3,45)_ = 1.41, *p* > 0.05, strategy initiation (*β* = −0.77, *p* < 0.05) and verbal concept formation (*β* = 0.71, *p* < 0.05) scores were significant predictors of the criterion variable at this level.

### Accuracy ratio: overall task performance

We hypothesized that overall IQ might predict overall task accuracy and completion rates and entered Full Scale IQ scores of the WASI into the regression model. The model was significant *F*_(1,45)_ = 9.11, *p* > 0.001, *β* = 0.41, *p* = 0.001 (*r* = 0.41, *p* = 0.01) indicating the important contribution of general intelligence to overall task completion accuracy. In addition, on the basis of correlation data, findings showed that Discrepancy (prospective plan implementation) and Overall task accuracy ratio scores constitute CT variables that show the most consistent relationship with EF and IQ variables across difficulty levels, although the conventional caveats should be borne in mind when interpreting correlation data. Except for the easy level, there was a consistent association between prospective memory, Verbal IQ and Discrepancy score although the direction of this relationship, and contribution of predictors to the criterion variable was different as task difficulty increased across levels, arguably suggesting shared processing resource costs across these variables as a consequence of increased task difficulty. Overall accuracy ratio scores showed a moderate relationship with Full Scale IQ on the basis of correlation data.

## Discussion

The present study investigated whether a newly developed interactive computerized CT functioned as an ecological measure of executive processes and captured similar functions as current off-the-shelf standardized tests in a normal group before trials with TBI cohorts. The CT had four difficulty levels (easy/moderate/difficult/dual-task) and findings indicated that each level had different processing demands likely due to the additional cognitive load required for difficult and dual-task levels. We expected CT variables to be associated with specific subtests of standardized tasks rather than global or overall scores because we designed CT variables with the intention of tapping into very specific processes likely recruited during real world CT. An expected finding was that executive function subtest measures predicted CT variables; however, an unexpected finding was that the relationship between executive function predictor and CT criterion variables differed as an effect of difficulty level based on regression analyses.

Thus, rather than difficulty level making increased demands on the same processes, findings of regression analyses indicate that the moderate and difficult levels of our task difficulty actually recruited different cognitive resources. This finding is a useful cautionary note because it indicates that unless test designers carefully evaluate the processes contributing to varying levels of task difficulty, as we have done here, it might be wrongly assumed that standardized task measure the same cognitive processes to greater or lesser degree rather than task demands actually initiating the implementation of different processes as a function of task difficulty.

Several variables predicted the CT strategy implementation measure (Range score) including scores on measures of prospective memory, performance-based IQ and executive function measures of concept formation and rule detection comprising verbal and performance-spatial based processes. The contribution of these variables to the CT variable differed for each difficulty level. Findings suggest that at the easy level strategy implementation on the CT was driven by non-verbal performance based reasoning, but as task difficulty increased strategy implementation depended more on prospective verbal-based planning and application of performance based planning strategies. At a broader level these findings challenge current conceptualizations of executive function because rather than overarching “executive” functions governing task-based activity on “real-world” tasks, findings suggest fluidly organized processes incorporating verbal and non-verbal working memory processes and strategy based executive functions that correspond well to the notion of a fractionated and *malleable* executive function system (Roca et al., 2014), but also indicate a central role of working memory and general intelligence to performance on tasks thought to depend primarily on executive functions (Royall and Palmer, 2014).

Verbal IQ and event-based prospective memory predicted the prospective plan implementation (Discrepancy score) measure of the CT. Prospective memory contributed to performance on the easy level but again as task demands increased verbal IQ was the unique predictor of prospective plan implementation. This finding indicates that at the dual-task level of our task capacity to draw on effective verbal reasoning is the key component for switching between the two tasks and implementing an effective plan. Again, whilst switching is typically defined as an executive function, again our findings indicate a key role of verbal IQ to prospective planning on the CT.

For the CT planning accuracy was quantified as time spent making mid-plan adjustments to items in order to achieve the end goal within the given time frame. Previous research using real-life CT have shown normal individuals to make significantly fewer errors (often zero) compared to those with frontal pathology (Godbout et al., 2005; Chevignard et al., 2008) and due to our non-pathological cohort, some ceiling effects on this measure were found. However, the adjustment scores also showed promise by indexing standardized cognitive measures, with our analyses showing that executive function measures of flexible thinking, accurate planning implementation and rule detection, predicted cross-level planning accuracy. Again, the relationship between predictors and the criterion variable differed as an effect of increased task difficulty based on regression results. Flexible thinking was an important mediator of plan accuracy at the easier levels and rule detection capacity predicted plan accuracy at the dual-task level. Overall, findings indicated that at all levels plan accuracy on the CT made similar demands on planning functions indexed by standardized executive function tests.

This version of the CT incorporated modifications from an earlier design to capture residual time; amount of time remaining from the tasks prescribed time limit and a measure of overall task accuracy. Rule detection and verbal concept formation were found to be significant predicators of residual time score towards for easier CT levels although strategy initiation was a significant predictor for three of the four levels including the dual-task level. Again, as with other CT measures, results indicated that that the more difficult levels general verbal based strategy processes contributed to task efficiency.

Finally, percentage accuracy ratio measured performance ability across levels of the CT based upon number of failures occurring at overall CT task and/or level. Full Scale IQ scores significantly predicted this criterion and arguably suggest that the CT distinguished between executive function and intelligence contributions to performance by showing selective executive function contributions to certain task components, but a key contribution of IQ to overall task accuracy. The notion that performance on executive function and IQ measures depends upon shared processes is a key debate in the literature (Royall and Palmer, 2014) and our preliminary findings indicate that CT measures can effectively distinguish between executive functions and FSIQ abilities. However, one limitation of our present findings is that regression analyses showed only moderate contribution of standardized neuropsychological predictor variables to CT criterion variables. The strong relationship between overall IQ score and overall accuracy on the CT task arguably suggest that overall intelligence is a crucial factor in task accuracy on this cooking measure. It is likely that both performance and verbal IQ contribute to effective task completion because sequential ordering of start and stop times for cooking items is likely mediated by a verbal plan of execution, with performance IQ contributing to spatial and non-verbal components of cooking ability. This area represents a further line of enquiry with real-world executive function analog tasks, with future research comprehensively distinguishing between non-verbal performance and verbal IQ contributions to successful task completion.

Overall, findings indicated that several standardized IQ, memory and executive function subtests predicted performance on the CT indicating that our real world simulation of an everyday activity reliably captured these functions in a normal cohort. The pattern of relationships between variables differed as a consequence of task difficulty and the use of a secondary task. Of note, in the present study all but one of the participants passed the secondary task (table setting was programed for pass/fail outcome only) suggesting the possibility of an accuracy trade-off across the primary cooking task activities and the secondary task.

Although at an early stage of development, the relationships found between CT indices and standardized measures holds great promise for the use of the CT as an ecologically valid measure of executive function. An updated version of the task, applicable to a greater number of platforms is currently under development and we hope to utilize this version in a TBI population to better understand the functions contributing to real world abilities, improve the predictive utility of clinical assessment and inform strategic rehabilitative approaches.

## Conflict of interest statement

The authors declare that the research was conducted in the absence of any commercial or financial relationships that could be construed as a potential conflict of interest.
